# Medico-Legal Aspects of Hospital-Acquired Infections: 5-Years of Judgements of the Civil Court of Rome

**DOI:** 10.3390/healthcare10071336

**Published:** 2022-07-18

**Authors:** Michele Treglia, Margherita Pallocci, Pierluigi Passalacqua, Giuseppe Sabatelli, Lucilla De Luca, Claudia Zanovello, Agostino Messineo, Giuseppe Quintavalle, Alberto Michele Cisterna, Luigi Tonino Marsella

**Affiliations:** 1 Department of Biomedicine and Prevention, University of Rome “Tor Vergata”, 00133 Rome, Italy; michele.treglia@uniroma2.it (M.T.); pierluigi.passalacqua@students.uniroma2.eu (P.P.); lucilla.deluca.09@students.uniroma2.it (L.D.L.); claudia.zanovello@students.uniroma2.eu (C.Z.); agostino.messineo@uniroma2.it (A.M.); marsella@uniroma2.it (L.T.M.); 2 Centro Regionale Rischio Clinico, Regione Lazio, 00145 Rome, Italy; gsabatelli@regione.lazio.it; 3 Fondazione Policlinico “Tor Vergata”, 00133 Rome, Italy; giuseppe.quintavalle@ptvonline.it; 4 XIII Sezione, Civil Court of Rome, 00192 Rome, Italy; alberto.cisterna@giustizia.it

**Keywords:** hospital-acquired infections, nosocomial infections, costs of nosocomial infections, medical liability

## Abstract

Introduction: Healthcare-associated infections (HAIs) represent a risk to patients’ health, as well as being an issue of worldwide relevance in terms of public health and increased healthcare costs. The occurrence of a complication causally related to the development of an infection contracted during a hospital stay, or in any event during a healthcare activity, may represent a source of liability for the healthcare facility itself and, therefore, lead to compensation for the injured patient. The aim of this research is to analyze the phenomenon of professional liability related to HAIs, to emphasize its economic and juridical aspects and, at the same time, highlight the clinical-managerial issues deserving attention, in order to guarantee the safety of care for patients. Methods: The retrospective review concerned all the judgments regarding HAIs drawn up by the Judges of the Civil Court of Rome, published between January 2016 and December 2020. Results: In the five-year period considered, 140 verdicts were issued in which the liability for which compensation was sought was related to the occurrence of healthcare-related infections. Convictions were recognized in 62.8%. The most involved branches were those related to the surgical areas: orthopedics, heart surgery, and general surgery. The three most frequently isolated organisms were Staphylococcus aureus, Pseudomonas aeruginosa, and Klebsiella pneumoniae. The total amount of compensation paid was EUR 21.243.184,43. Conclusions: The study showed how the analysis of the juridical and medico legal aspects of HAIs may represent not only a helpful tool for healthcare performance assessment, but also a data source usable in clinical risk management and in the implementation of patient safety.

## 1. Introduction

It has been estimated that, globally, there are approximately 1.4 million people constantly experiencing hospital-acquired-infection-related complications [1].

In the past, infections associated with hospitalization were defined as “nosocomial infections” but, since the 1990s, with the proliferation of outpatient healthcare services, an extension of the concept of outpatient care assistance and social health was needed.

Hospital-acquired infections (HAIs) can be defined as the most common viral, bacterial, and fungal origin infections or other uncommon pathogenic diseases occurring while receiving treatments for medical or surgical conditions in healthcare settings (such as hospitals, outpatient clinics, dialysis centers, long-term care facilities, home healthcare services, and territorial public health authorities) and which were not present or were incubating at the time of admission [2]. They also include those clinical cases in which symptoms of infections appear after discharge, and those affecting the medical staff, thus representing occupational infections [3]. From a chronological point of view, according to the definition of the European Centre for Disease Prevention and Control (ECDC), a hospital-acquired infection can be defined as an infection occurring within 48 h of hospital admission and within three days of discharge [4].

HAIs may include infections transmitted via external sources (exogenous), such as person-to-person contact or infected healthcare workers or settings, and infections caused by bacteria present in body sites (endogenous), such as saprophytic bacterial flora in the skin or intestine.

From the etiological point of view, the most frequent HAIs are those related to invasive medical devices, including central line-associated bloodstream infections (CLABSIs), catheter-associated urinary tract infections (CAUTIs), ventilator-associated pneumonia (VAP), and surgical site infections (SSIs) [5].

With regard to the most involved microorganisms, although trends vary according to the different national realities (and over years), it has been shown that bacteria belonging to the Enterobacteriaceae family, such as Acinetobacter Baumanii and Pseudomonas Aeruginosa, are currently the most commonly isolated, while among Gram-positive bacteria, Staphylococci, especially Methicillin-resistant Staphylococcus aureus (MRSA) and Enterococci are the bacteria detected in the highest numbers [6,7].

The Italian Ministry of Health estimates that, yearly, infections occurring in hospitalized patients range from 450,000 to 700,000 (a healthcare-associated infection occurs in around 4–7% of hospitalizations) [8]. An Italian cross-sectional study carried out in 2016 in some urgent care units revealed that the incidence was 1296 HAIs among 1186 patients [1]. It has been estimated that in Europe, HAIs, each year, cause: 16 million additional days of hospitalization, leading to a direct increase in the length of stay (LOS); 37,000 cases where HAIs were leading causes of death; and 110,000 cases where HAIs were contributory causes of death. The estimated costs are around EUR 7 billion, including only direct costs [9]. The situation observed in the United States is equally concerning: 2 million HAI cases are diagnosed annually, with 90,000 deaths (the onset of HAIs is the fifth leading cause of death in urgent care units). Compared with other causes, HAIs are the most common leading cause of clinical complications, with a rate ranging from 5% to 10% of patients involved and rising costs of around USD 4 billion [10]. The substantial importance of the above-mentioned numbers clearly points out that HAIs represent a problem with crucial impacts on public health assistance costs, as well as clinical risk management. Indeed, the different incidence of nosocomial infections may be used as a quality-of-care index. Furthermore, in this context, in cases of causation-related complications leading to the onset of an infection during hospitalization or healthcare care service provision, they may constitute, under certain circumstances, grounds for professional liability of the healthcare facility in question, leading to compensation for damage to the patient, with rising costs for public and private health facilities and insurance companies. Despite the remarkable increase in the amount of medical malpractice claims around the world, however, it has been a poorly considered and investigated aspect in the scientific literature [11,12]. Fault-based liability may arise in specific cases, such as non-fulfillment of specific hygiene and aseptic procedures during health service provision; underestimation of immunocompromised patients or patients with specific pre-existing comorbidities (diabetes mellitus, chronic diseases, intake of immunosuppressive drugs); poor sanitation of the operating rooms or improper asepsis of surgical instruments; or, more generally, non-compliant healthcare facility conduct with all the scientifically validated precautions designed to prevent any occurrence of infection. In terms of preventability, it was highlighted that in medical settings equipped with specific infection control and surveillance protocols managed by expert epidemiologists, the infection rate was 32% lower than in healthcare settings lacking the above-mentioned protocols [5]. Even more recent studies have shown that up to 55% of HAIs are preventable [13]. Furthermore, it must not be forgotten that, hypothetically, a considerable number of patients might have suffered from permanent damage or even have died from potentially preventable infections. Therefore, considering the multiplicity and variability of the conditions that may facilitate the onset of an infection during hospital stays, and their ascertained partial preventability, forensic evidence of the occurred events on fault-based medical liability following nosocomial infections may be extremely complex, although crucial, in legal proceedings. The purpose of this paper is to report the data obtained from the analysis of the judgments on medical malpractice-related infections issued by the Civil Court of Rome, the main court at the national level by number of litigations, representing around 20% of all national ones.

## 2. Materials and Methods

The retrospective review covered all judgments written by the Judges of the Civil Court of Rome, XIII Section, published between January 2016 and December 2020. Only first instance judgements were taken into consideration, excluding both second instances, as well as those issued by the Supreme Court of Cassation. The XIII Section of the Civil Court of Rome deals with professional liability trials, including the medical field. The University of Rome “Tor Vergata” and the Civil Court of Rome signed an agreement, for which the court provided the judgments for analysis. The research was initially performed using the keywords “medical liability” and “medical professional”. The documents were saved in PDF format and anonymized to preserve litigants’ personal identities and remove any connection between the tort in question and specific individuals or institutions. At the end of the anonymization phase, out of 1190 total documents (of which 23 duplicates were deleted), only 1167 underwent a preliminary analysis, performed by three different auditors skilled in medical professional liability, which led to a further exclusion of 50 documents not referable to medical negligence issues, but rather concerning, more specifically, veterinary and car accident liability.

**Figure 1:**
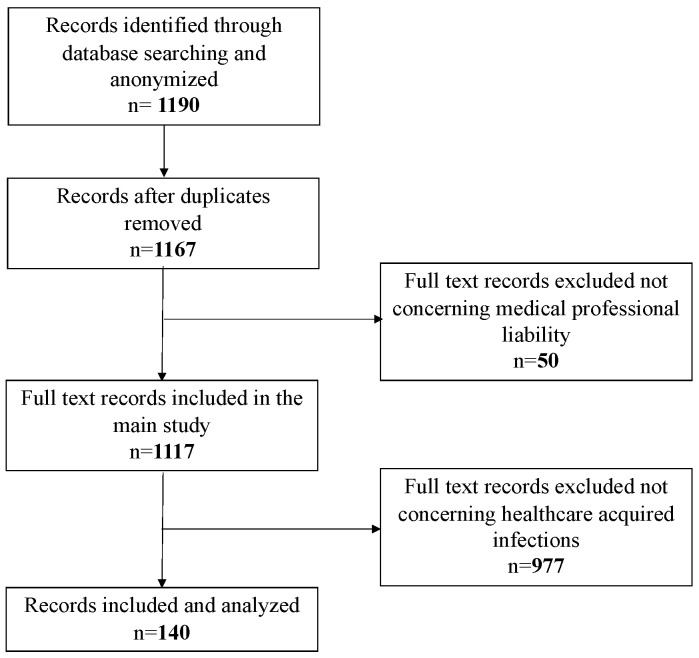
Flowchart reassuming the documents process selection.

The second step involved the analysis of 1117 documents exclusively relating to medical malpractice cases. For the analysis, a work grid was used to process the data, using the EXCEL program (Office 365), to systematize the data mining. The grid was also set up with some locked fields, to minimize the inter-individual variability between the three auditors. The items present in the columns of the excel grid were: judgment no., occurrence year, publication year of the judgment, medical specialty involved, type of negligence/liability sued and recognized, type of damage (injury/death), type of parties involved (public/private facility or single healthcare worker), outcome of the trial, and compensation paid. At the end of this step, 140 judgments concerning medical liability cases with underlying healthcare services–related infections (Figure 1) were investigated. The latter were further analyzed in order to detect both the type of microorganisms involved and the related infection, when expressly mentioned in the judgment.

It should be noted that some fields of the grid could not be filled in because the required information was not available, so they were left empty, but this did not affect the reliability of our study. Finally, the filter function of the EXCEL program was used to process and cross-reference the data.

## 3. Results

**Figure 2:**
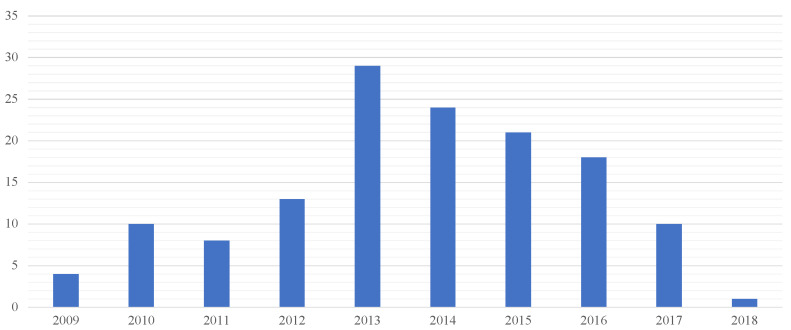
Number of Judgments introduced per year.

In the five-year study, 140 compensation claims referred to healthcare-acquired infections damage (that is 12.5% of the total of judgments in the medical professional liability field issued by the court over the period in question). The judgments analyzed (issued during the five-year reference period 2016–2020) concerned lawsuits introduced in the period from 2009 to 2018, most of which were introduced in 2013–2014 (Figure 2).

**Figure 3:**
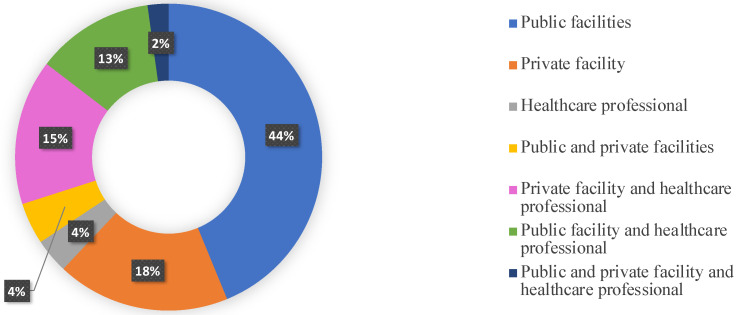
The parties involved.

In 60 of 140 (42.9%) cases, only public health facilities were involved; in 25 of 140 (17.9%) cases only private facilities were involved; in 41 of 140 (29.3%) cases, both physicians and health facilities (public > private) were sued (Figure 3). Only in 5 out of 140 (3.6%) cases was a healthcare professional arraigned, specifically, in dentistry (4/140) (2.9%) and in neurosurgery (only one case).

Convictions as proof of the existence of a direct or indirect HAI-related professional liability were recognized in 88 out of 140 (62.8%) litigations, a higher rate if compared to the total number of convictions analyzed over the period in question, in which professional liability was ascertained in 55% of cases.

**Figure 4:**
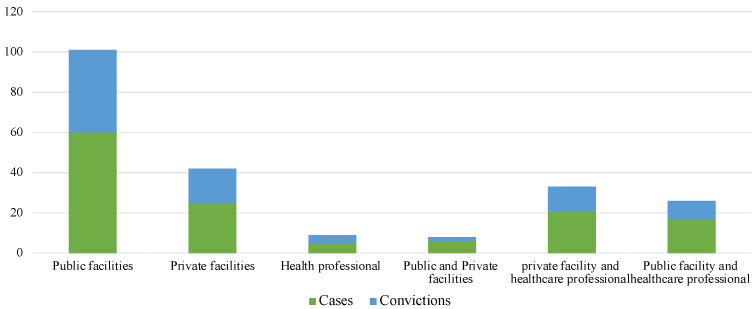
Numbers of cases and related convictions, distinguished for the four branches most involved.

Sixty cases involving only public health facilities showed that they were recognized liable and sentenced to award compensation in 68.3% of cases; in private facilities, sentences were issued in 64% of cases. On the other hand, the conviction rate rose to 57.1% in cases involving both healthcare professionals and private facilities, to drop to 52.9% in those cases in which only the healthcare provider and one or more public facilities were sued (Figure 4).

**Figure 5:**
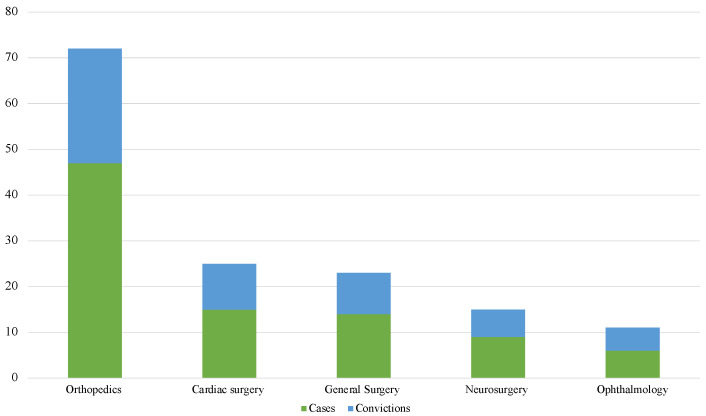
Branches most involved.

The most involved branches were those related to the surgical areas (Figure 5). Orthopedics were involved in 47 of the 140 (33.6%) cases, followed by heart surgery with 15 out of 140 (10.7%) cases, and general surgery with 14 out of 140 (10%) litigations. With regard to the data related to the risk of conviction diversified by branch, sentences resulted in 25 out of 47 (53.2%) of orthopedics cases, in 10 out of 15 (66.6%) of cardiac surgery cases, and in 9 of 14 (64.3%) of those relating to general surgery. Particularly interesting is the data relating to ophthalmology, representing 6 out of 140 (4.3%) cases, with a litigation/conviction ratio of 83.3%.

As for the damage, death was disputed in 45 of 140 cases (32.1%) and 95 of 140 cases (67.9%) concerned non-fatal injuries. With particular reference to the latter, 83 out of 95 of them (87.4%) concerned mild or serious surgical site infections (involving contiguous or distant tissues) and 5 out of 95 (5.3%) cases were HCV infections acquired during hospitalization and leading to hepatitis (all sentenced). A further 3 out of 95 (3.2%) sentences were about the same lawsuit of a nurse suffering from active tuberculosis; the remaining 4 cases involved different types of damage (endocarditis, vulvovaginal infection following the in vitro fertilization -IVF procedure). On the other hand, as regards deaths, in all cases they were associated with pulmonary or urinary sepsis, while only one case concerned CLABSI.

**Figure 6:**
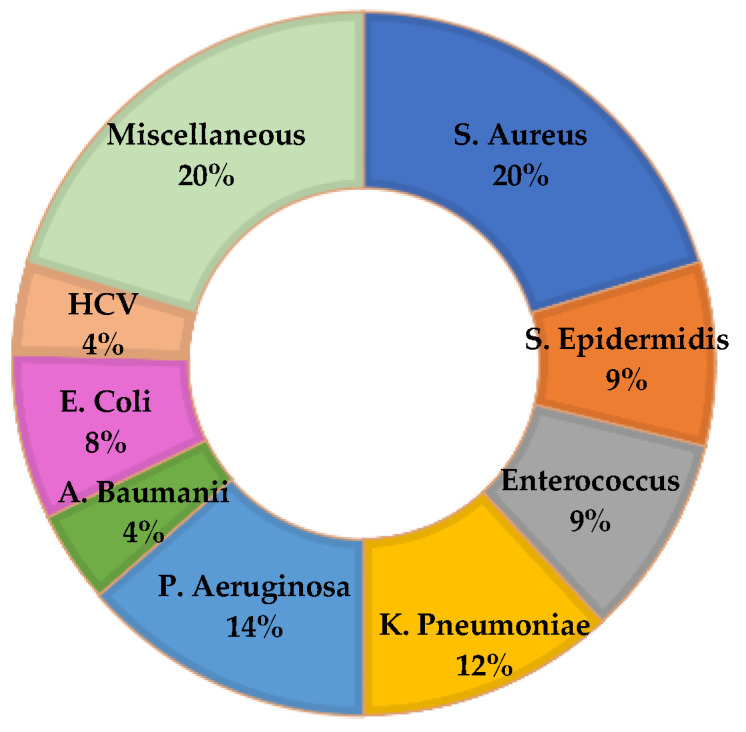
Microorganisms involved in HAIs.

Microorganism-related judgments were specifically reported only in 83 out of 140 (59.3%) cases: in 53 out of 83 (63.8%) the infection resulted from a single species of microorganism, and a polymicrobial origin was found in 30 out of 83 (36.1%) cases. The three most frequently isolated organisms were Staphylococcus aureus, Pseudomonas aeruginosa, and Klebsiella pneumoniae (Figure 6). In only one case, Kocuria Kristinae was isolated. This is a microorganism belonging to the family of gram-positive cocci, widely present in the environment and often found in the common skin and mucous bacterial flora of humans and other mammals. Poorly described in the scientific literature as a human pathogen, it is the microorganism mostly associated with systemic infections in immunocompromised patients or in subjects with vascular catheters. Our case, however, concerned a hip arthroplasty infection, in a healthy subject.

Over the entire period under investigation, the total amount of compensation paid (excluding legal charges and property damage) was EUR 20,255,463.11. The maximum compensation paid to an immunocompromised patient suffering from myelodysplastic syndrome was EUR 1,483,268.15: the clinical conditions of the patient, suffering from P. Aeruginosa-related infection, progressively worsened, and culminated in sepsis, MOF, and finally death (Table 1).

## 4. Discussion

The Italian Ministry of Health have focused specific attention on healthcare-related infection issues with the introduction of ministerial circulars (no. 52/1985 and no. 8/1988), laying down coordinated measures for nosocomial infection prevention, active surveillance, and control, which are not always translated into consistent, extensive, effective and homogeneous actions across the national territory. Subsequently, various specific documents on HAI surveillance were issued by the above-mentioned Ministry of Health, such as the Compendium of Strategies to Prevent HAIs, along with recommendations for the control of nosocomial spread of methicillin-resistant Staphylococcus aureus (MRSA) or relating to the prevention of other infectious diseases with significant impacts on public health, such as measles, rubella, HIV, tuberculosis, and vector-borne diseases. Some of these recommendations may also be found in the National Prevention Plan 2014–2018 and in the National Action Plan on Antimicrobial Resistance (PNCAR) 2017–2020, which further emphasize the need and the importance of effective measures for the prevention and control of infectious diseases and antibiotic resistance. In particular, insofar as it is of interest, the aforementioned documents set out several requirements, such as the identification of regional authorities for HAI surveillance, the implementation of the national surveillance action plan, and the establishment of a rapid alert organism system for the detection of the onset of epidemic clusters and significant events within healthcare facilities. The importance of surveillance and prevention measures and, in general, of a proper application of clinical risk management standards, have played a more prominent role after the introduction of Law no. 24/2017, a regulatory act by which the Italian Legislator has placed great emphasis on patient safety-related issues, stating in Article 1 that “safety must be guaranteed through proper prevention tools and health care risk management, in conjunction with the most effective use of structural, technological and organizational resources available”. Healthcare professionals must contribute to risk prevention while administering health care procedures, thus pointing out the need for coordinated and integrated intervention at all levels.

The central role played by prevention in the field of healthcare associated infections has been further highlighted as a consequence of the lack of the above-mentioned measures, allowing the identification and definition of a potential fault-based medical liability. Currently in Italy, with regard to civil liability, a distinction is required between healthcare facilities’ liability and healthcare providers’ liability, causing a drastic rise in costs for healthcare facilities, which are called upon to demonstrate both the correctness of their conduct and that of their healthcare employees [14]. It is clear that, with regard to nosocomial infections, the burden of proof, mostly on facilities, has been worsened by the fact that facilities, public or private, represent the extensive totality of the cases concerning HAIs, being the only potentially liable parties in court, a clear trend emerging even from the judgments we analyzed. In addition, it is well known that the view expressed by many jurists about the fact that, in the field of medical liability, the need for stricter measures specifically applicable to HAIs (a phenomenon impossible to eradicate but to some extent preventable), is based on politics of law theories more than on specific dynamics regulating civil liability. With respect to the above, the following excerpts are from the analyzed HAI-related litigation judgments, showing the criteria for determining criminal liability of facilities, as well as forensic evidence considered indispensable for the exemption of guilt: “As for the measures adopted in terms of prevention to contrast nosocomial infections, it should be noted that, for the exclusion of criminal liability, the facility must adequately document to have complied with the guidelines and best practices on the subject of asepsis …. the burden of proof is on the hospital in question that is required to prove that it has diligently observed and predisposed all the necessary measures to ensure the safety of the hygienic conditions and the correct sterilization of all surgical instruments, in order both to meet all statutory requirements under the regulations in force and under the leges artis, and to prevent the onset of bacterial infectious diseases, relating to therapeutic treatments administered to the patient by healthcare providers, following the contraction of the infection …”.

The excerpts reported, besides emphasizing the importance of preventive measures with regard to health issues and medico-legal aspects, allow us to better understand the reasons underlying the higher number of convictions for litigations on medical negligence matters for the total amount of judgments under investigation (61.4% vs. 55%).

In this regard, a criticality that emerged from the analysis performed is related to the lack of a preventive measures standard to refer to. Most of the judgments based on generic precautions under the regulations in force and the leges artis state the prevention of the risk of infectiousness without mentioning any specific standard in favor of the burden of proof. It should also be pointed out that, in order to be found not liable, the health facility must document not only the identification of specific preventive operational recommendations but also, first and foremost, their strict implementation. In this regard, healthcare authorities willing to contrast HAI phenomenon, even at the judicial level, are asked to guarantee not only a proper environmental sanitization but also the collection, certification, and conservation of all documents potentially helpful for an eventual defense in court. Generally speaking, from a preventive point of view, different measures may be applied to minimize infectious diseases spread within hospitals, including, but not limited to: hand washing, sterilization, disinfection, isolation protocols, a wiser use of antibiotics, and standard of care relating to an adequate professional training and knowledge. Two of the simplest and potentially effective measures to adopt are hand washing and hospital staff education. However, these measures, although simple and strongly recommended, often, are not correctly performed, thus threatening the health and safety of patients. According to an example reported in a paper published in 2003 by an eminent scientific journal, the observance of hand washing in hospitals ranges from 16% to 81% [15] a wide variability affecting both patients’ health and, as discussed above, judgment outcomes The data reported show that the surgical area is the most involved with 54% of cases concerning only three branches: orthopedics, cardiac surgery, and general surgery. These data, substantially in line with what is reported in the scientific literature [16,17,18], confirms the data relating to SSIs’ incidence, representing the subject matter of the litigations in 93 out of 140 cases (including both fatal and non-fatal injuries) for a total of EUR 8,782,343.41 of compensation paid. With reference to the health costs deriving from SSIs, it is estimated that about 60% of cases might be prevented by complying with the existing guidelines, that patients were exposed to a risk of death 2 to 11 times higher, and that over 70% of these deaths were specifically attributable to SSIs. Additionally, the consequential average length of stay exceeded 10–14 days. For these reasons, SSI prevention has received considerable attention from all healthcare professionals involved in infectious diseases surveillance. It is also necessary to consider the impact that SSIs may have on public opinion, which is often considered as a quality-of-care index. As for SSI prevention, several guidelines and recommendation documents have been published and made available for consultation. Their difference lies in the different degrees of importance given to any single recommendation. By way of example, recommendations on antibiotic prophylaxis, trichotomy, and maintenance of normothermia are substantially similar for the WHO, NICE, and CDC. As for the control of glucose homeostasis and skin preparation techniques, they are similar, but they are based on using different degrees of force for different elements within the recommendations. Oral antibiotics are recommended in colorectal surgery along with mechanical bowel preparation by both the WHO and CDC, but not by NICE. These and further differences in the recommendations contained in the various guidelines may have an important impact on both the clinical management of patients and, as far as is of interest, in the medico-legal assessment in court cases, considering that, under Article 5, paragraph 1 of Law no. 24/2017, the healthcare professionals—whatever the purpose of their medical service: preventive, diagnostic, therapeutic, palliative, rehabilitative, or forensic—must comply with the recommendations provided by the guidelines or, failing those, by the clinical assistance standard, without prejudice to the peculiarities of the case in question. As shown by the reported data, HAI litigation management-related costs, taken as a whole, may have a significant impact in terms of financial resources’ allocation for public health expenditure, considering that, in Italy, most of the health facilities are public (being also those most frequently involved in HAI-related litigations).

Many studies have focused on HAIs’ severity and patients’ safety-related risks, trying to analyze their economic impact using different methods. These methods are often invalidated by the impossibility of trying to distinguish the type of treatment, and the total costs paid for the infection, from those for the pre-existing disease leading to the patient’s hospitalization. It is important to focus not only on the HAI’s incidence as a measure of effectiveness, but also on the events arising from its occurrence, such as antibiotic use, length of stay, mortality, and costs. Costs can be expected to be higher in tertiary referral hospitals. Costs may also differ across countries and over time. A British study found that, yearly, 320,994 patients contract an infection during hospitalization and that these infections exacerbate the National Health System expenditure by GBP 930.62 million [19]. Another work documented that for each confirmed case of infection, attributable costs ranged from USD 9310 to USD 21,013 with an LOS ranging from 5.9 to 9.6 days and with an attributable death rate of 6%. Total health care costs (for 159 patients) ranged from USD 1.48 to 3.34 million, and the cost attributable to premature deaths was USD 5.27 million [20].

According to our analysis, more than EUR 21,000,000 was paid out over 5 years, excluding compensation paid before trying judicial proceedings, with respect to which no data are available. However, as reported in the introduction, according to scientific literature, about 30% of HAIs are preventable, which makes it possible to hypothesize, starting from the data obtained, a potential saving of at least EUR 7,000,000; resources that might be effectively invested in procedures aimed at preventing HAIs, implementing patient safety, and, at the same time, reducing costs in terms of insurance premiums and compensation.

## 5. Conclusions

The study carried out showed how the analysis of judicial cases of medical liability may represent not only a helpful tool for healthcare performance assessment, but also a data source usable in clinical risk management and in the implementation of patient safety. In this perspective, a standardization of the assessment criteria at national level, with the involvement of each single health authority, would be advisable. In fact, from the “no blame” point of view, claims would represent for the facility a mirror of the assistance-related issues specifically in the HAIs’ field, leading to optimized financial resources and targeted interventions, aimed at reducing those specific adverse events related to potential litigations. A centralized data collection at the national level concerning HAI-related claims would certainly be helpful to identify the best-performing measures implemented by any single facility in terms of reduction in claims, with an undoubted advantage for preventive medicine.

## Figures and Tables

**Table 1:** Compensation paid in the period of investigation.

Total Amount Paid for HAIs (2016–2020)	Mean	Minimum	Maximum	Total Amount for Non-Fatal Injury	Total Amount for Fatal Injury	Total Amount for SSIs (Evolved or Not in Fatal Injury)
**EUR 21,243,184.43**	EUR 241,399.82	EUR 2074.50	EUR 1,483,268.15	EUR 3,912,013.6 (19.3%)	EUR 16,343,449.6 (80.7%)	EUR 8,782,343.41

## Data Availability

Not applicable.
